# Tobacco Use Among Rural Schoolchildren of 13-15 Years Age Group: A Cross-Sectional Study

**DOI:** 10.4103/0970-0218.69281

**Published:** 2010-07

**Authors:** Rekha P Shenoy, Prashanth K Shenai, Ganesh Shenoy Panchmal, Shashidhar M Kotian

**Affiliations:** Department of Community Dentisty India; 1 Department of Oral Medicine & Radiology, Yenepoya Dental College, Yenepoya University, Nithyananda Nagar P.O., Deralakatte, Mangalore - 575 018, India; 2 Department of Community Medicine, Kasturba Medical College, Mangalore - 575 003, India

## Introduction

An estimated 186 million of the world population are school children of 13-15 years. Among them, approximately 34.8 million are current tobacco users.(1)

In India, the most susceptible time for tobacco use is during adolescence and early adulthood (15-24 years). In rural settings, family members and neighbours who often ask young children to get tobacco from nearby shops. Media advertisements and colorfully packaged tobacco products act as pro-tobacco influences.(2)

There is an urgent need to curb tobacco use among youth. Hence, this study was conducted to estimate the prevalence of tobacco use among rural school children of 13-15 years and to find the reasons for use of tobacco products. Also, reasons for initiation, access, availability, source of funding, knowledge about the dangers of tobacco consumption, tobacco use among family and teachers, and cessation behavior were assessed.

## Materials and Methods

This study was undertaken from January to March 2009 in Deralakatte in Dakshina Kannada district of Karnataka State, India. Of the seven co-education schools here, three were selected by simple random sampling.

### Survey administration

Approval was obtained from the Institutional Review Board and heads of the schools after apprising them about the survey.

### Questionnaire

A 24-item structured close-ended questionnaire was used for data collection. It was pre-tested and reliability was assessed using Cronbach’s alpha internal consistency coefficient.

After explaining the study purpose and giving instructions for completion, it was administered to students and collected back after scrutinizing for completeness. Then, researchers answered students’ queries regarding tobacco use and its adverse effects.

### Statistical analysis

Data were analyzed using the SPSS Version 14.0. Differences in proportions between genders were compared using Chi squared (χ^2^) test. A difference was considered to be statistically significant if the *P* -value was <0.05. The Cronbach’s alpha value averaged 0.82.

## Results and Discussion

### Coverage

Sample consisted of 360 boys (58.1%) and 260 girls (41.9%); response rate [89.9%] was higher than other studies.(3,4)

### Knowledge of tobacco and tobacco products

75.3% boys and 75.8% girls knew about tobacco (χ^2^=0.0197; *P* =0.8884); 42.2% boys and 32.7% girls were able to name different forms of tobacco. (χ^2^=5.8076; *P* =0.016, significant)

### History of tobacco use

Tobacco use classification as “past use” (use in past, now discontinued) or “current use” (use in last 30 days, either daily/occasionally) are given in Tables 1 and 2.

**Table 1:** Distribution based on tobacco use by respondents

History of tobacco use	Boys *n* (%)	Girls *n* (%)
Past tobacco use	12 (3.3)	2 (0.8)
Current tobacco use	64 (17.8)	19 (7.3)
Never used	284 (78.9)	239 (91.9)

Total	360 (100)	260 (100)

(χ^2^=22.251; *P*<0.001, very highly significant)

**Table 2:** Distribution based on forms of tobacco used by current users

Form of tobacco used	Boys *n* (%)	Girls *n* (%)
Smoked form (cigarette, beedi, etc.)	11 (17.2)	3 (15.8)
Smokeless form (pan, gutka, etc.)	44 (68.8)	15 (78.9)
Both forms (cigarette, beedi, pan, gutka, etc.)	9 (14)	1 (5.3)

Total	64 (100)	19 (100)

(χ^2^=4.154; *P*=0.125)

**Table 3:** Distribution based on reasons for initiation of tobacco use among current users

Reason	Boys *n* (%)	Girls *n* (%)
Use by friends	11 (17.2)	3 (15.8)
Use by family	8 (12.5)	7 (36.8)
For style/fashion	6 (9.4)	0 (0)
Peer pressure	3 (4.7)	2 (10.5)
No specific reason	36 (56.2)	7 (36.9)

Total	64 (100)	19 (100)

(χ^2^=9.213; *P*=0.109)

Current use was more among boys.(2,5–8) Others found little difference between genders.(4,9) Some studies found higher prevalence of current users;(1,4,6,7) and others the reverse.(3,5,8) Current smokeless tobacco use was more common than current smoking.(4,6,8,9) Reverse has also been recorded.(1,3,5) A small proportion used both forms.(6)

### Duration of use among current users

37.5% boys reported three-month use, 18.8% reported use for two years and 18.8% reported use for more than two years. Respective values for girls were 47.4%, 21.1% and 10.5%. (χ^2^=1.27; *P* =0.8664)

### Frequency of use among current users

32.8% boys reported once daily use, 4.7% had a daily usage of five or more times. Comparative figures among girls were 58% and 10.5% respectively. (χ^2^=16.49; *P* =0.0024, highly significant)

Table 3 lists the reasons for initiation of tobacco use among current users.

### Access to and availability of tobacco among current users

19.4% boys and 17.3% girls reported availability near homes and schools. (χ^2^=0.456; *P* =0.499) 73.4% boys and 78.9% girls had not been refused purchase despite being minors.(1,5,6) (χ^2^=0.235; *P* =0.627)

### Source of funding for tobacco habit among current users

53% boys and 68.4% girls used their pocket money.(2) (χ^2^=3.574; *P* =0.3112)

### History of tobacco usage among family and teachers

34.4% boys and 35.4% girls reported tobacco usage within their family.(3,5,6) (χ^2^=0.058; *P*=0.808) The father was most commonly named.(6) Tobacco use by teachers was reported by 6.7% boys and 2.3% girls, (χ^2^=6.229; *P*=0.0126, significant) less compared to GYTS.(6)

Table 4 lists awareness of the dangers of tobacco consumption. Findings were similar to other studies;(2,7,10) some found lower awareness levels.(5,6) Major sources of information were media, school teachers and doctors/dentists.(2)

**Table 4:** Distribution based on respondents’ knowledge of whether tobacco use is harmful

Response	Boys *n* (%)	Girls *n* (%)
Yes	305 (84.7)	252 (96.9)
No	55 (15.3)	8 (3.1)

Total	360 (100)	260 (100)

(χ^2^=24.617; *P*<0.001, very highly significant)

### Effect of tobacco use on self-esteem of current users

Increased self-esteem was reported by 25% boys and 26.3% girls.(2) (χ^2^=0.013; *P*=0.907)

### Problems due to tobacco use among current users

Cough, oral malodor and stained teeth were the complaints of 40.7% boys and 68.4% girls. (χ^2^=25.783; *P*=0.007, highly significant)

### Desire to quit tobacco use among current users

60.9% boys and 31.6% girls expressed a desire to quit (χ^2^=5.087; *P*=0.021, significant); 43.7% boys and 36.8% girls had tried quitting the previous year. (χ^2^=0.286; *P*=0.592) Other studies reported higher values.(1,3,4,6,10)

### Social support

30.1% current users had received tobacco cessation advice from health personnel (χ^2^=0.0249; *P*=0.8746); figures lower than GYTS data.(6) Only 6.1% boys and 3.5% girls said they would offer pro-tobacco advice to siblings/peers. (χ^2^=2.231; *P*=0.135)

### Limitations of study

(1) small sample; (2) data applicable to those who attended school on day of survey; therefore, not representative of all 13-15-year-olds; (3) data based on self-reports (may have under-/over-reporting of behavior).

To conclude, rising tobacco use among schoolchildren is a disturbing reality and strict enforcement of tobacco policies is a dire necessity.
